# “Surgery is Thinking”: cognitive neuroscience perspective for the AI age

**DOI:** 10.3389/fsurg.2026.1769343

**Published:** 2026-04-02

**Authors:** Ronan A. Cahill, Pol MacAonghusa, Mindy Nunez Duffourc

**Affiliations:** 1 Department of Surgery, Mater Misericordiae University Hospital, Dublin, Ireland; 2 Centre of Precision Surgery, University College Dublin, Dublin, Ireland; 3 Maastricht Law and Tech Lab, Maastricht University, Maastricht, Netherlands

**Keywords:** artificial intelligence, decision-support, extended mind thesis, liability, surgery, surgical video, minimally invasive surgery, robotic assisted surgery

## Introduction

With advancements in minimally invasive surgery (MIS), surgical video has evolved from a historical record to an integral component of the operation, closely intertwined with the surgeon’s actions. Any integration of artificial intelligence (AI) further transforms surgical video into a dynamic element that can influence the surgeon in real time during the operation (1). Such innovations are intended to enhance surgical capability via augmented and potentially automated operative components (2). However, AI-adapted surgical video may introduce new risks, especially because intraoperative decision–action intervals are short and consequences are often irreversible. This article examines the transcending role of surgical imaging through a cognitive neuroscience lens to consider differently the clinical and medico-legal implications of AI-augmented perception in surgery, rather than the traditional view of surgical video and, indeed, AI as just tools.

## Cognitive neuroscience perspective of surgeon perception and misperception

**Figure 1:**
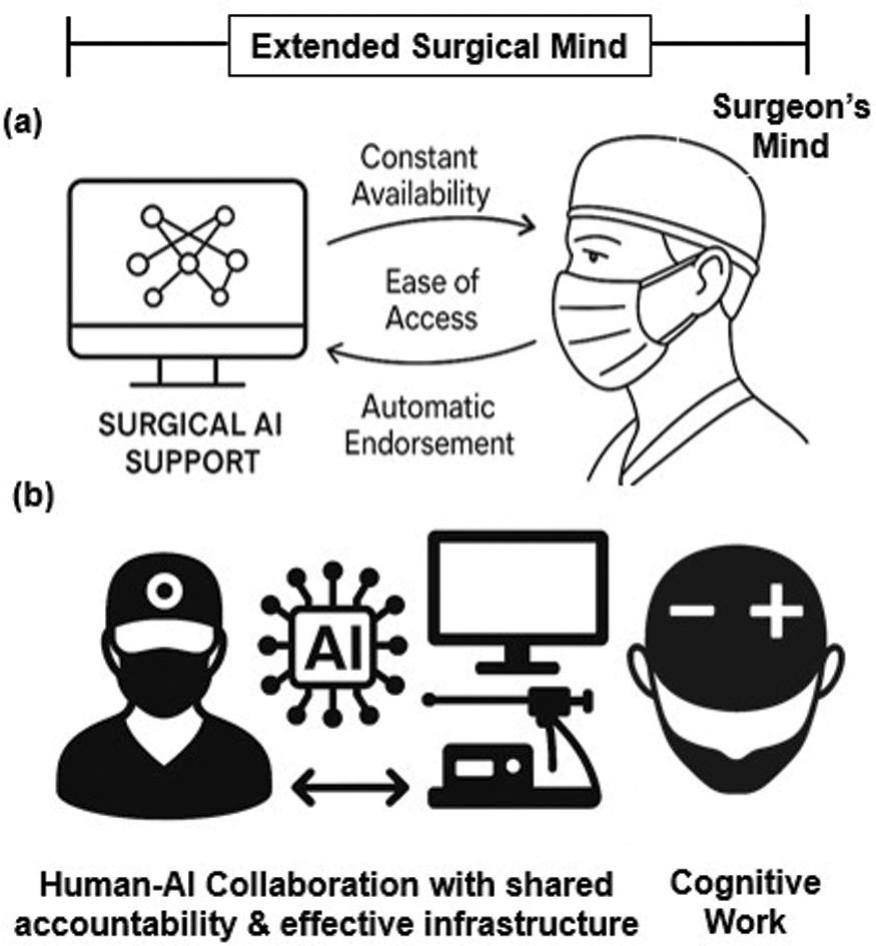
Humans frequently use aids for problem-solving. These aids become part of cognition when they perform functions typically carried out in the “head” and meet certain criteria, including constant availability, ease of use, and direct useability, with outputs that are automatically endorsed **(a)** In such coupled systems, the external component becomes crucial to cognition (removing it can reduce cognitive competence in a manner analogous to losing part of the brain). Surgical AI decision-support systems can meet these criteria and are therefore distinct from similar systems used postoperatively (e.g., for video analysis). Surgical extended-mind systems need therefore **(b)** augment human cognition by (i) being trustworthy and user-friendly to alleviate mental strain (rather than increasing it by providing excessive or irrelevant recommendations), (ii) share responsibility and accountability for errors with clinicians and other components of the system framework (e.g., data feeds), as suboptimal environments may inhibit perception, and (iii) operate with seamless integration **(b)**.

Humans interact with reality through perception, which includes neural inputs from their *a priori* knowledge and experience. In MIS, surgical insight emerges only when operative video, serving as sensory input, is transformed by the surgeon's perceptual processing into scene pattern recognition and meaningful interpretation (the surgical camera's digitalization of the internal operative scene into a stream of pixels represents structured but inert signals until they are interpreted). Like all perception, surgical perception is a predictive process, in which the surgeon's cognition repeatedly generates hypotheses to reconcile against incoming sensory signals. During operations, the surgeon's own well-trained mind, including anatomical expectations, judgement, and previous experiences (both positive and negative), and heuristics, is essential for processing video display imagery with the speed and capacity required to make sequential decisions under uncertainty. Under the “extended mind” theory (Figure 1), surgical equipment, including video feeds, can therefore be viewed as extensions of the surgeon's mind (3). Operative interpretations are therefore not created in the surgeons' eye or mind but arise in combination with the surgical video imagery, which serves as the substrate for the cognitive scaffold that the surgeon uses to reason. This is equivalent to the concept that “writing is thinking” (4) or to solving a mathematical equation on a chalkboard—where the writing tool and canvas are intrinsic to the cognitive process.

While surgical expertise enhances surgeons' perception and cognitive capacity for surgical decision-making, surgeons (like all humans) are susceptible to cognitive illusions that can cause misperception (5). These can stem from interpretation biases, such as attentional and confirmation blindness, ambiguity resolution, and expert “overconfidence,” which can cause a surgeon to see what they expect to see rather than what is actually present. Such well-recognized limitations of human perception in other high-risk industries, including aviation, have led to the development of systems (such as slowing down, checklists, or double verification for pilots) that counterbalance flawed heuristics to prevent human error (5). Perioperative checks and surgical timeouts are current examples; however, until now, there has been little systematic verification of intraoperative surgical decisions. Digital assurance of perfusion sufficiency or identification of critical anatomy using near-infrared fluorescence-guided surgery is increasingly accepted as one such adjunct paving the way for AI-augmented surgical video. Notably, the tradition of implicit confidence in surgeon's decisions meant that such technologies arguably faced greater resistance and required more rigorous evaluation than dexterity-enhancing innovations such as robotic assistance (6, 7).

## Role for intraoperative AI in surgery today

In addition to representing reality to the surgeon, surgical video also generates data amenable to computational and AI analysis. Some AI systems that work on surgical video data in real time are simply surgical tools that act via passive externalism (i.e., digitally reducing cautery smoke that obscures the surgical field). Other AI systems directly serve as partners in decision-making at critical stages of the surgery (i.e., active externalism) (2). When AI systems enter this space and become tightly coupled with the surgeon's decision-making processes, they become part of the “Surgical Extended Mind.” An approach that treats all AI applications in surgery as “just another tool” without recognizing this important distinction underappreciates both the potential risks and benefits of AI in surgery. On the other hand, acknowledging that AI can affect a surgeon's consciousness and behavior (such as the surgeon becoming dependent on or uncritical of the AI) enables the development of approaches that mitigate these risks, including those arising from poorly trained or deliberately manipulated algorithms, and define accountability for AI systems.

## Exbodiment of surgical thinking

The integration of artificial intelligence into surgical decision-making may also be interpreted through the lens of *exbodiment*, a developing theoretical framework in cognitive science and theoretical biology that extends embodiment and extended-mind perspectives, emphasizing the inseparability of perception, action, and environment (8, 9). Within this view, cognition is not confined to neural processes alone but can be partially outsourced to engineered matter, such that external artifacts function as computational collaborators and co-evolve with human cognition as components of a single adaptive system. Organisms are understood to construct niches that compensate for biological constraints; in this sense, AI systems may be regarded as technologically constructed cognitive niches that enable surgeons to mitigate limitations of perception, memory, and bias. The long-term aim of surgical AI integration would therefore not be the replacement of human judgment but the emergence of support systems sufficiently fluid that their use approaches incorporation into skilled action. Consistent with extended-mind theories, repeated interaction with external tools can lead to the internalization of their operational logic, reflected neurally in a shift from effortful prefrontal engagement toward premotor and sensorimotor representations as expertise develops. Notably, exbodiment also emphasizes the productive role of constraint: imperfect or limited tools can reorganize cognition by prompting novel strategies and new forms of problem solving. Interaction with imperfect but informative AI systems may therefore foster new modes of surgical reasoning, provided that responsibility, interpretability, and human oversight remain clearly structured.

## Medico-legal implications for AI as part of the surgeon's own cognition

AI-driven surgical technologies capable of influencing the cognitive processes that drive intraoperative decision-making may affect the legal standard of care used to assess surgeons' liability (see Supplementary Table S1). At its core, the standard of care evaluates whether a surgeon's decisions were objectively reasonable (10). As AI extends into the surgeon's cognitive process to aid decision-making, liability law may need to shift in its reasonableness assessment. On one hand, advanced AI systems that help surgeons overcome limitations of human perception may mean that misperceptions that were once considered reasonable could become unreasonable and lead to liability. For example, anatomical misperceptions that cause a surgeon to injure a patient's bile duct during laparoscopic cholecystectomy may constitute a breach of the standard of care if (1) AI technology capable of improving intraoperative dissection decision-making was available and (2) a reasonable surgeon would have used that technology. On the other hand, if AI systems influence a surgeon’s perception in ways that contribute to cognitive errors and lead to unreasonable surgical decisions, the surgeon may be liable for failing to realize these effects. In any case, intraoperative reliance on AI must be assessed for reasonableness, regardless of whether the AI-influenced decision proved correct (10, 11). Reasonable use of AI for cognitive support in surgery also requires that surgeons understand and mitigate its risks, including overreliance and automation bias. AI developers and healthcare organizations also share responsibility for the safe design and implementation of AI systems (and should bear liability when they fail).

## Surgical video recordings as objective evidence

Surgical video, like other data, can be recorded for later review, although it is typically not routinely incorporated into the medical chart at present (the surgeon's operative note as the summary of the surgical act takes this place). As the capability to store and process large amounts of data continues to expand, operative recordings are now seen as valuable data sources. However, if surgery is understood as embodied cognition in action, surgical recordings can be seen as external memory of the participants involved. Ascribing ownership and rights to such recordings becomes layered (attribution is important for legal discovery and, increasingly too, as a source for training and testing AI models). While the governing medical institution can claim ownership of video recordings captured through its resources and policies (similar to how medical records are treated), patients or surgeons (present via their actions derived from their perceptions, interpretations, and decisions) may also claim access or indeed credit and any usage is further restricted by personal privacy laws (such as the General Data Protection Regulation (GDPR) in the European Union). Furthermore, subsequent viewings, including analysis of operative perception and performance quality, introduce new cognitive inferences from new viewers, including hindsight bias and retrospective clarity when the outcome is known without the real-time perceptual limitations and pressures. Without proper appreciation, such retrospective evaluation can create a “liability trap.” While AI can provide re-analysis of operative recordings, we need to be careful not to allow it to retrospectively ascribe “missed” anatomy or errors that an unaided surgeon could not have realistically detected under normal human constraints (reasonable human misperceptions may not breach the standard of care, which demands reasonable, not perfect or superhuman, performance).

## Conclusion

Surgical cognition should therefore be understood as a distributed system spanning neural processing, technological mediation, and institutional responsibility. Recognizing intraoperative AI not merely as an instrument but as a component of the cognitive architecture reframes both surgical expertise and accountability. The future governance of intraoperative AI should therefore align technological design, clinical training, and legal standards with this emerging model of distributed intelligence.
