# Supplementary material for: “Surgery is Thinking”: cognitive neuroscience perspective for the AI age
Source: Front Surg. 2026 Apr 2;13:1769343. doi: 10.3389/fsurg.2026.1769343 (PMC13085633; doi:10.3389/fsurg.2026.1769343)
Supplement: Supplementary file 1 [file Table1.docx]

| **Era of Surgery** | **Dominant Cognitive Model** | **Role of Technology** | **Cognitive Relationship** | **Learning & Adaptation** | **Neural Integration** | **Typical Risks** | **Medico-Legal Framing** | **Representative Example** |
| --- | --- | --- | --- | --- | --- | --- | --- | --- |
| Open Surgery | Embodied cognition | Physical instrument | Tool external to cognition | Surgeon adapts to tool | Minimal integration beyond motor skill | Manual error, fatigue | Individual operator responsibility | Scalpel, retractors |
| Minimally Invasive Surgery (MIS) | Extended Mind | Perceptual interface | Technology forms cognitive scaffold | Reciprocal coupling between perception and display | Visual–motor remapping and perceptual learning | Misperception, attentional bias | Surgeon remains primary decision-maker | Laparoscopic video monitor |
| AI-Augmented Surgery | Surgical Extended Mind | Decision-support system | Active cognitive partner influencing judgement | Continuous feedback between surgeon and algorithm | Internalization of tool logic with expertise | Automation bias, overreliance | Shared influence but unclear liability boundaries | AI perfusion assessment or anatomy recognition |
| AI-Integrated Adaptive Systems (Emerging) | Exbodiment | Computational collaborator within constructed cognitive niche | Co-evolving human–technology system | Mutual adaptation across individual, technological, and institutional levels | Transition toward sensorimotor fluency and predictive reliance | Algorithmic bias, distributed error sources | Distributed responsibility across surgeon, developer, and institution | Real-time adaptive intraoperative AI guidance |

# Supplementary Table S1 Evolution of Surgical Cognition and Technological Integration The role of technology in surgery has evolved from external instruments supporting embodied skill toward cognitively integrated systems that participate in perception and decision-making. AI-assisted surgery may represent a transition from extended cognition toward exbodiment, in which human expertise, engineered systems, and institutional structures co-adapt within a distributed cognitive framework.
